# Poly(a) selection introduces bias and undue noise in direct RNA-sequencing

**DOI:** 10.1186/s12864-022-08762-8

**Published:** 2022-07-22

**Authors:** Marcus J. Viscardi, Joshua A. Arribere

**Affiliations:** Department of Molecular, Cellular and Developmental Biology, University of California at Santa Cruz, Santa Cruz, CA USA

**Keywords:** Oxford Nanopore, Direct RNA-sequencing, Poly(a) selection, RNA-sequencing, Transcriptomics

## Abstract

**Background:**

Genome-wide RNA-sequencing technologies are increasingly critical to a wide variety of diagnostic and research applications. RNA-seq users often first enrich for mRNA, with the most popular enrichment method being poly(A) selection. In many applications it is well-known that poly(A) selection biases the view of the transcriptome by selecting for longer tailed mRNA species.

**Results:**

Here, we show that poly(A) selection biases Oxford Nanopore direct RNA sequencing. As expected, poly(A) selection skews sequenced mRNAs toward longer poly(A) tail lengths. Interestingly, we identify a population of mRNAs (> 10% of genes’ mRNAs) that are inconsistently captured by poly(A) selection due to highly variable poly(A) tails, and demonstrate this phenomenon in our hands and in published data. Importantly, we show poly(A) selection is dispensable for Oxford Nanopore’s direct RNA-seq technique, and demonstrate successful library construction without poly(A) selection, with decreased input, and without loss of quality.

**Conclusions:**

Our work expands the utility of direct RNA-seq by validating the use of total RNA as input, and demonstrates important technical artifacts from poly(A) selection that inconsistently skew mRNA expression and poly(A) tail length measurements.

**Supplementary Information:**

The online version contains supplementary material available at 10.1186/s12864-022-08762-8.

## Background

Identification of RNAs in a biological sample is central to diverse research applications including mechanistic studies, diagnostics, and high-dimensional phenotyping. Several techniques of genome-wide RNA sequencing (RNA-seq) exist to survey the transcriptome, broadly falling into sequencing-by-synthesis (Illumina, 454, PacBio) or sequencing-by-current (Oxford Nanopore). But before RNAs can be sequenced, they must first be captured in a manner amenable to sequencing.

Biases inherent in RNA capture protocols can skew the view of the transcriptome (reviewed in [1]). Indeed, comparison of several RNA-seq techniques on identical samples demonstrates biases from each protocol [2]. These biases can arise at a number of steps, including: RNA fragmentation, reverse transcription priming, PCR amplification, and capture on the sequencing platform. Among transcriptomic techniques, the Oxford Nanopore Technologies (ONT) direct RNA-sequencing (dRNA-seq) platform stands out by avoiding nearly all of these steps, and a prior study detected less bias in Oxford’s dRNA-seq protocol compared to several others [2].

With the recent introduction of total RNA as input for ONT’s dRNA-seq protocol, the opportunity to avoid additional handling by omitting poly(A) selection has increased the utility of the technique, and provides the opportunity to directly answer questions regarding the potential biases introduced by the previous standard of oligo (dT)-based selection methods. In other sequencing applications, poly(A) selection artificially enriches for longer tailed RNA species and therefore would be expected to also bias the dRNA-seq technique [3–5]. Work from several groups highlights examples where poly(A)-tail lengths and deadenylation rates differ between mRNAs according to their age or gene-of-origin [6, 7]. An ideal dRNA-seq protocol would explicitly avoid biases inherent in poly(A) selection.

Here, by analyzing libraries with and without poly(A) selection, we show that the use of oligo (dT)-based poly(A) selection introduces bias and variability attributable to mRNA poly(A) tail differences. We extend our analyses to include samples from another lab to show that poly(A) selection introduces undue noise in published data. The omission of poly(A) selection minimizes poly(A)-tail-derived biases and allows for a substantial reduction of input sample RNA, enhancing the accuracy and expanding the applications of dRNA-seq.

## Results

### Omission of poly(a) selection enables lower input for ONT direct RNA sequencing

**Fig. 1:**
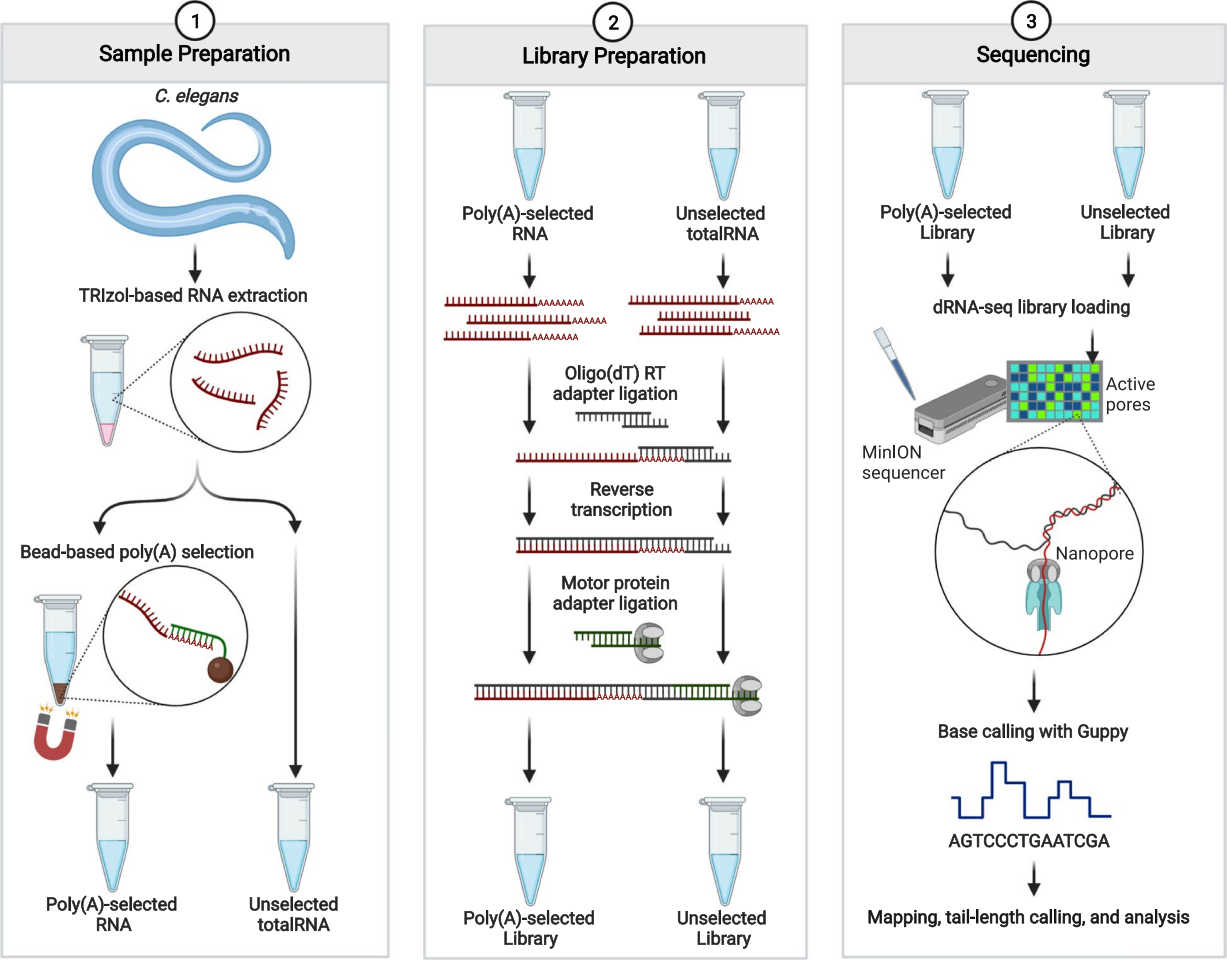
Workflow used in omission of poly(A) selection in ONT dRNA-seq protocol. Outline of procedures used for “selected” and “unselected” libraries analyzed in this study. Procedures detailed in the methods section. Figure created with BioRender.com

Due to the inherent requirement for a short sequence of 3′-terminal adenosines during the oligo (dT) splint based ligation in ONT dRNA-seq (Fig. 1), we reasoned that there would still be specificity for mRNAs in the ONT protocol without additional poly(A) selection. To ensure an adequate number of poly(A)-tailed RNAs available for sequencing, we changed the input to ONT dRNA-seq from 500 ng of poly(A)-selected RNA (selected from ~ 50-100μg total RNA) to 5 μg of total RNA. Updates to the ONT dRNA-Seq protocol since the preparation of these libraries now allows for inputs as low as 50 ng of poly(A)-selected RNA, or 500 ng of total RNA.

We produced five ONT dRNA-seq libraries from two independent biological samples: three libraries with poly(A) selection (hereafter called “selected”) and two without poly(A) selection (hereafter “unselected”) (Table 1). (We also included synthetic mRNAs of known poly(A) tail length in these libraries, but these standards failed for technical reasons and thus will not be discussed further.) Despite the > 10-fold reduction in starting material, our poly(A)-unselected libraries yielded only ~ 30% less reads on average (Table 1). We expect that future optimization of dRNA-seq with total RNA input will improve read counts. In both our poly(A)-selected and -unselected libraries, a high fraction of all reads mapped to protein-coding genes, demonstrating that dRNA-seq of total RNA produces similarly successful libraries (Table 1 and further investigated in Fig. 3).

**Table 1:** Selected and Unselected Libraries

Library Name:	Bio Sample	Total RNA Mass into Selection	RNA into Library	Flow Cell #	Hours On Flow Cell	Guppy Reads Called	minimap2 Primary Reads Mapped	Reads Assigned to Protein Coding	Bases Called and Mapped
Selected-1	A	90.0 μg	464 ng	7	63 hours	1779.5 k	1754.6 k	1097.7 k	1465 Mb
Unselected-1		–	5.0 μg	8	63 hours	1345.5 k	1234.6 k	820.3 k	1030 Mb
Selected-2	B	44.8 μg	349 ng	3	23 hours	1388.2 k	1344.5 k	909.7 k	1063 Mb
Unselected-2		–	5.0 μg		39 hours	759.8 k	702.2 k	484.5 k	567 Mb
Selected-3		90.0 μg	500 ng	1	67 hours	2017.1 k	1972.6 k	1287.5 k	1748 Mb

**Fig. 2:**
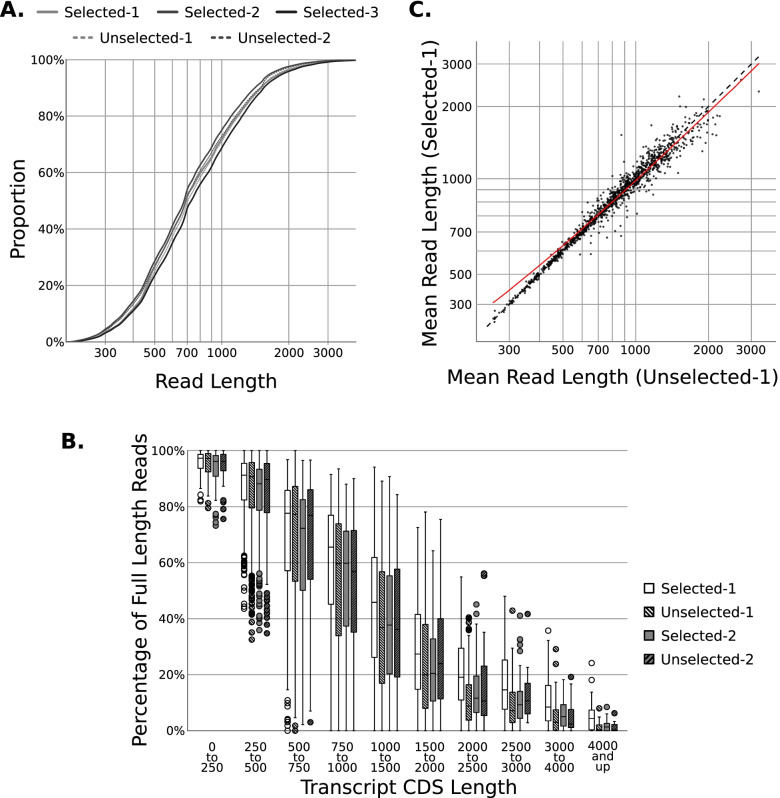
Omission of poly(A) selection does not significantly alter read lengths from ONT direct RNA-seq. **A** Cumulative distribution function of basecalled read lengths from three poly(A)-selected libraries, and two libraries in which poly(A) selection was omitted. This plot contains all basecalled reads, including those which were not mapped via MiniMap2. Later plots only include mapped reads. **B** Genes with only a single annotated transcript were binned based on their CDS length, then reads mapping to that gene were denoted as “spanning-CDS’ or “not-spanning-CDS” based on the location of the 5′ and 3′ read ends. Finally the percentage of “spanning-CDS” reads were calculated and plotted for each gene.﻿ **C** Plot comparing mean read lengths of each gene between a poly(A)-selected library (Y-axis) and an unselected library (X-axis) (Spearman R: 0.9713)

Library read lengths are a common metric of success for ONT dRNA-seq runs, as overhandling of RNA or introduction of exogenous RNases will lead to shorter reads. All libraries had similar read lengths, with an overall mean length of 985 nucleotides and no significant difference between poly(A)-selected and -unselected runs (Paired T-test: *p*-value = 0.3115) (Fig. 2A, Table 1). Reasoning that a subtle bias in read length would be missed from this analysis, we analyzed reads lengths stratified by gene CDS (Coding Sequence) length, and again we saw no significant difference between the libraries (Fig. 2B). The libraries’ mapped read lengths were also highly similar on a gene-by-gene basis, with spearman correlation coefficients of 0.98 and 0.97 (Fig. 2C). Based on these analyses, we conclude that omission of poly(A) selection does not significantly impact read lengths, but does allow for a reduction in starting material for dRNA-seq libraries.

### Omission of poly(a) selection leads to marginally reduced transcriptome complexity

**Fig. 3:**
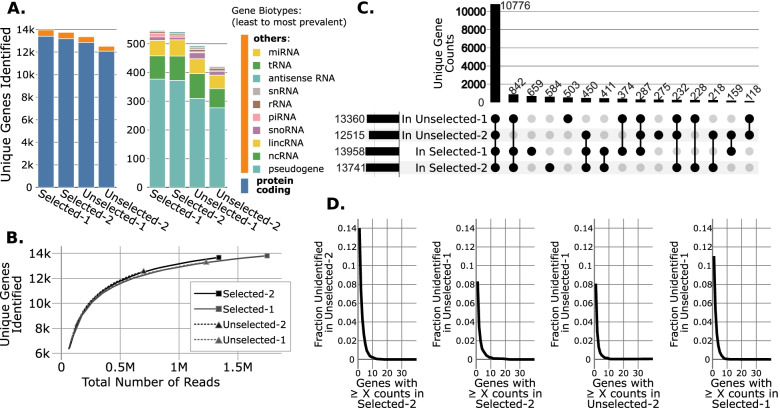
Unselected libraries capture slightly less complex transcriptomes due to their reduced read depth. **A** Comparison of the number of unique genes identified (having 1 or more reads mapped) and their biotypes in each library. The right plot zooms into the “other” (orange) biotypes from the left plot. **B** Saturation analysis of the number of unique genes identified in each library. Each line indicates the mean of 100 repeated subsamples for each integer percentage of the overall number of reads in that library. Standard deviation of the mean at each subsample point was also plotted but is not visible at this scale. **C** Upset plot [8] showing the coordinance of genes identified in each library. The bars at the bottom left of the plot indicate the number of genes identified in each library (as shown in Subfigure A and B). The bars at the top of the plot indicate the number of unique genes identified by the subset of libraries indicated by the filled circle below. **D** Inverse CDF plots showing the fraction of unique genes having more than “X” read(s) in the X-axis library that were unidentified (having zero reads mapped) in the Y-axis library. The four comparisons shown each reached an unidentified fraction of zero at X = 16, 22, 42, and 17, respectively (indicating all of the genes in the X-axis library with more than “X” reads we identified in the Y-axis library)

Transcriptome complexity analyses are common methods of assessing the utility of a sequencing technique to identify a biologically significant portion of the transcriptome. We quantified the number of unique genes captured and their RNA biotypes for each library (Fig. 3A). We observed no major changes in the biotypes captured, but we observed a slight reduction in the number of unique genes identified in the unselected samples. We hypothesized that this reduction arose from the reduction in sequencing depth of unselected libraries. To test this hypothesis, we subsampled each library and saw that when total read count was equal, selected and unselected libraries had identical numbers of unique genes identified (Fig. 3B).

To assess if genes that were identified in each library were similar, we performed an overlap analysis (Fig. 3C). Many genes (10,776) were identified in all four libraries (Fig. 3C). While several hundred genes were missed in one or more libraries, this number was consistent across different library types and samples, arguing against wholesale loss of genes in selected or unselected libraries (Fig. 3C). Our subsequent analyses showed that many genes are lost in one or more libraries due to their low abundance, as expected (Fig. 3D). Taken together, these analyses show that there is a small loss in transcriptome complexity from omission of poly(A) selection, and that this effect is largely due to the global reduction in library depth (Table 1).

### Poly(a) selection underestimates expression of mRNAs with short poly(a) tails

**Fig. 4:**
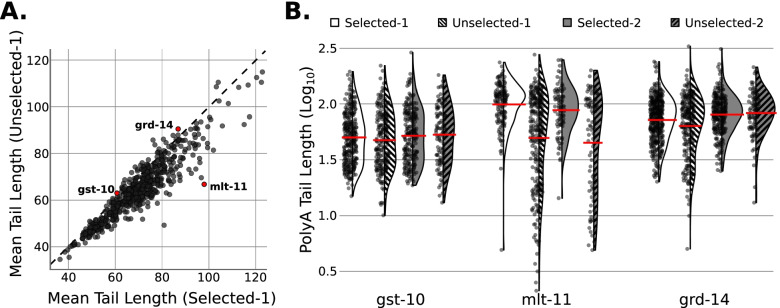
Omission of poly(A) selection captures shorter-tailed RNA species. **A** Scatter plot comparing mean tail lengths called by Nanopolish PolyA for each gene between selected and unselected libraries for Replicate 1. Dashed black line indicates the diagonal where tail length in both libraries is equal. Genes of interest shown in subfigure 4B highlighted in red. **B** Violin plots showing distributions of poly(A) tail lengths called by Nanopolish PolyA for genes highlighted in subfigure 4A. Red horizontal lines indicate the mean tail length. Kernel density estimation completed using default settings of Plotly python package (version = 5.3.1)

Given the expectation that poly(A) selection would preferentially recover longer poly(A) tails [3–5], we analyzed poly(A) tail lengths across the libraries. We estimated poly(A) tail lengths for each mRNA molecule using Nanopolish [9]. Aligning with our expectation, the vast majority of genes’ mRNAs had longer mean tail lengths in selected libraries compared to unselected libraries (Fig. 4A). Some genes’ mRNAs showed no significant change in mean poly(A) tail length between the selected and unselected libraries (e.g., *gst-10* & *grd-14*, Fig. 4B), while others exhibited dramatically shorter tail lengths in unselected libraries (e.g., *mlt-11*, Fig. 4B). In general, genes with dramatic changes in their mean tail length had a subpopulation of shorter tail-length reads that were only captured in unselected libraries. This observation is expected based on the biochemistry of the poly(A) selection method: mRNAs with short poly(A) tails would inefficiently bind oligo (dT) and thus be lost during poly(A) selection [4, 5]. The splint adapter in dRNA-Seq is not expected to suffer this same bias as the annealing and ligation can only occur at one position (the 3′ end of the transcript) provided enough adenosines exist for splint binding.

**Fig. 5:**
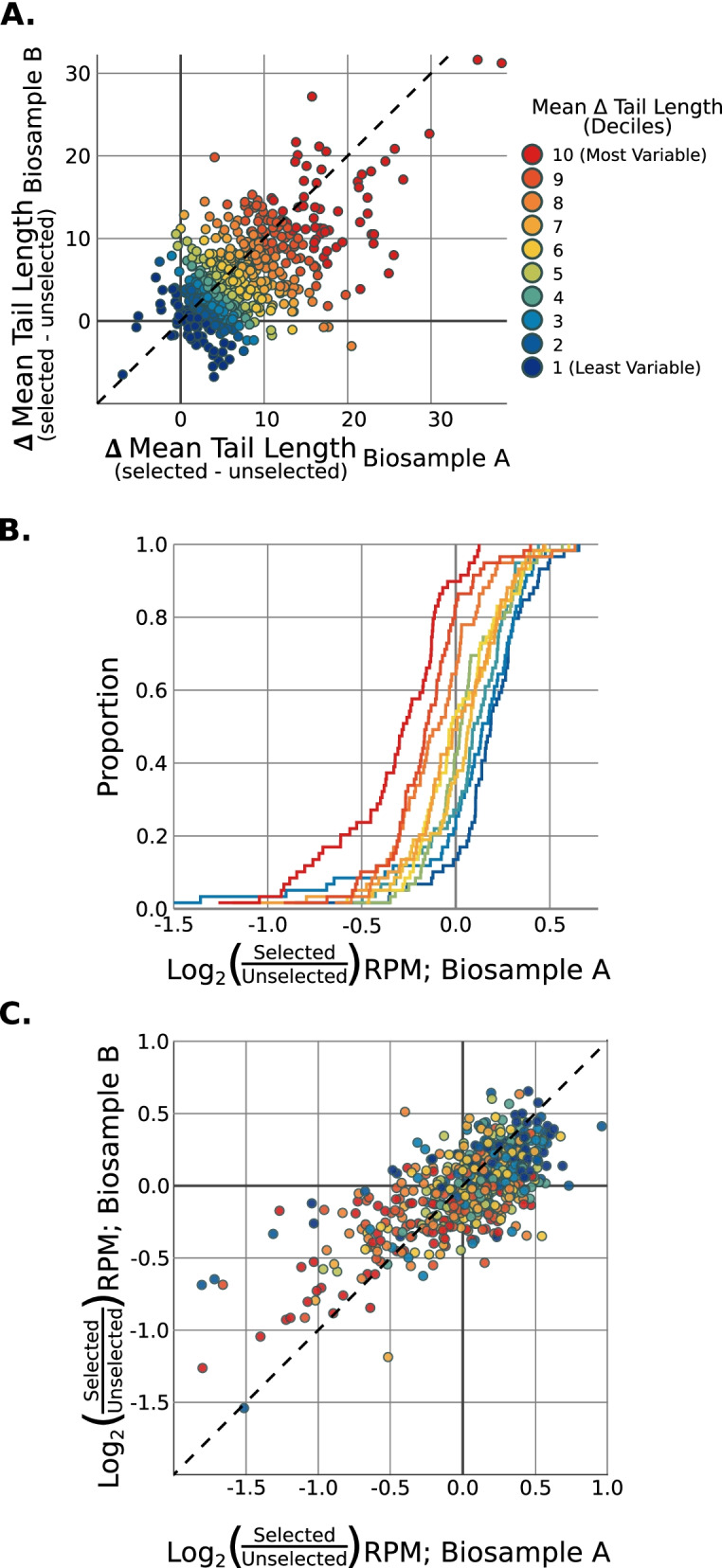
Poly(A) selection underestimates expression of genes w/ short-tailed mRNAs. Two biological replicates were used in these subfigures to identify systemic technical biases (Selected-1 & Unselected-1; Selected-2 & Unselected-2). **A** Scatter plot comparing the differences in Nanopolish-called mean tail length between techniques (selected mean tail length - unselected mean tail length). Each axis is from one biological sample. Genes are colored based on decile binning of the average difference in tail lengths between techniques. Dashed line indicates the diagonal, where the difference in tail lengths is equal. **B** Cumulative distribution functions of the fold-change RPM between techniques. Genes were broken up into deciles of average difference in tail lengths between techniques and replicates as shown in Fig. 5A. **C** Scatter plot comparing fold-change RPM between techniques for two biological samples, to assess reproducibility of technical biases. Genes are again colored by decile as per Fig. 5A. Dashed line indicates the diagonal where the fold-change in RPM due to technique is equal between biological replicates (Spearman correlation coefficient: 0.6422)

In addition to bulk skewing of poly(A) tail lengths, selection for longer poly(A) tails could also lead to differential capture of mRNAs and skew the transcriptome. We thus identified genes with mRNAs exhibiting a large difference in mean poly(A) tail lengths in poly(A)-selected samples compared to unselected samples (e.g., *mlt-11*, Fig. 4B; Fig. 5A). For simplicity, we call such genes “variable tail length genes”. Variable tail length genes were consistent across biological replicates (Fig. 5A). Upon examination of the mean expression of genes’ mRNAs (in RPM, as defined in Methods), we found that variable tail length genes were preferentially lost upon poly(A) selection (KS Test results: Replicate 1: *p*-value = 1.3E–11; Replicate 2: p-value = 5.3E–15) (Fig. 5B, C). Thus poly(A) selection causes gene-specific recovery biases, which could easily be misinterpreted as apparent “changes” in the number of mRNAs expressed from a gene.

### Poly(a) selection bias is variable across replicates

**Fig. 6:**
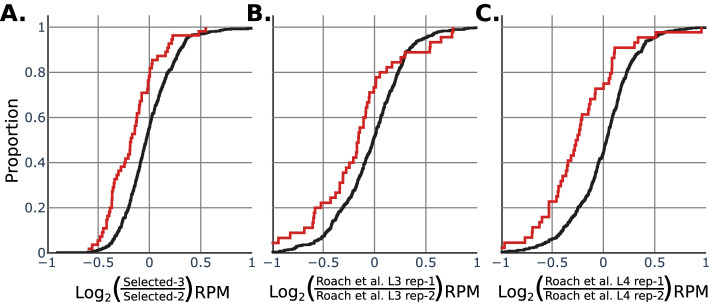
Poly(A) selection is a source of variability in technical replicates. Cumulative distribution functions of the fold-change RPM between replicates. Each plot is a comparison of two polyA-selected library replicates performed on the same biological sample. The red line in each denotes genes that fell in the most variable decile of tail lengths, shown as red in Fig. 4A (decile 10). Black lines denote all other genes, falling into deciles 1 through 9. **A** Comparison of two technical replicates produced for this study: Selected-2 and Selected-3. (KS test, decile 10 vs decile 1–9: *p*-value = 0.00018848) (**B** and **C**) Comparison of two sets of poly(A)-selected technical replicate libraries from Roach et al., 2020 [10]; ENA public accession numbers: roach_L3_1: ERR3245468, roach_L3_2: ERR3245469, roach_L4_1: ERR3245470, roach_L4_2: ERR3245471 (Roach L3 set: KS test, decile 10 vs decile 1–9: p-value = 0.00519075. Roach L4 set: KS test, decile 10 vs decile 1–9: p-value = 0.00007283)

Variability in the poly(A) selection bias could also confound interpretation of poly(A)-selected libraries, adding unnecessary noise to sample replicates. Indeed, comparing technical replicates of poly(A) selection in our hands, we found that variable tail length genes were differentially captured (KS Test: *p*-value = 0.000328) (Fig. 6A). To determine whether the effect would manifest in datasets from other labs, we performed the same analysis on two sets of similarly-staged *C. elegans* dRNA-seq libraries from Roach et al. 2020 [10] (ENA accession: PRJEB31791) (Fig. 6B and C). In both pairs of replicates, we observed that genes with highly variable tail lengths were differentially captured compared to all other genes (KS test: L3 stage sample *p*-value = 0.00535, L4 stage sample p-value = 0.000002). These results demonstrate that poly(A) selection adds unnecessary noise to the quantification of gene expression as read out by Nanopore dRNA-seq.

## Discussion

Here, we evaluated the requirement for poly(A) selection in Oxford Nanopore Technologies’ direct RNA sequencing protocol. We showed that the poly(A) selection step is unnecessary for the assurance of dRNA-seq library success.

Even in successful poly(A) selections with no detectable difference in captured fragment lengths, we observed artifacts. First, we observed preferential capture of longer tailed mRNAs globally. Global changes in the captured RNA pool are a blind spot for many differential analyses, which assume the mean/median genes’ mRNAs do not change. Thus we recommend caution when interpreting dRNA-seq data generated from poly(A)-selected RNA populations. Second, we observe inaccurate capture of genes that produce mRNAs with variable tail lengths, an effect which led to inconsistent capture of these genes’ mRNAs in our and others’ hands. These results support the idea that poly(A) selection introduces unnecessary noise to differential expression analyses, and skews the view of the transcriptome. Our work echoes a report from *S. cerevisiae* [11], where ~ 800 of ~ 6000 genes were differentially captured in selected vs. unselected libraries. We note that due to the oligo (dT) splint ligation inherent to ONT dRNA-seq, the dRNA-seq protocol will still be unable to capture a subset of the transcriptome, including pre-mRNAs, deadenylated degradation intermediates, and tail-less mRNAs (e.g., histone mRNAs).

## Conclusions

Our results demonstrate that poly(A) selection can lead to poly(A)-tail-dependent capture biases. Such capture biases could be misinterpreted as differential gene expression. Most RNA-seq applications make inferences from the population of captured transcripts (e.g., expression levels, splicing analysis, poly(A)-site usage, promoter usage), and our results demonstrate that inclusion of poly(A) selection can skew the captured transcripts. Our work in *C. elegans* echoes an earlier report using samples from *S. cerevisiae* [11]. Given the biases and noise introduced by poly(A) selection, we recommend omission of poly(A) selection, despite a modest loss in statistical power and transcriptome complexity from decreased read counts. Further optimization of the dRNA-seq protocol for use with total RNA may also solve the decreased read count problem.

## Methods

### Strains

A single N2 *C. elegans* strain was used for all sequenced libraries (VC2010, [12]). For all preparations, *C. elegans* were grown at 20C on NGM (nematode growth medium) plates using OP50 as a food source.

### RNA collection

Animals (*C. elegans*) were bleached to obtain a synchronous population of eggs, and then grown at 20C for 44 hours. Animals were collected on a sucrose cushion to minimize bacterial contamination, pelleted, and washed with EN50 and M9. Pellets were resuspended in TRIzol (Ambion, cat#15596026), lysed by freeze-cracking, and total RNA was isolated by chloroform extraction. Total RNA integrity was assessed with the Agilent high-sensitivity RNA system for TapeStation. For later analysis and sequencing, only total RNA samples with RNA integrity number equivalent (RINe) values greater than 7.0 were used.

### Poly(a) selection

Perkin Elmer NEXTFLEX Poly(A) Beads (2.0) were utilized for selection of polyadenylated mRNA species based on the manufacturer’s specifications. Briefly, total RNA was run over magnetic beads pre-bound to oligo (dT) to anneal polyadenylated RNA, then washed and eluted to isolate mRNA. Inputs and reaction size were chosen to yield at least 500 ng of Poly(A) RNA for ONT dRNA-seq. Tapestation was used to confirm the loss of 18S and 28S ribosomal RNA.

### Library preparation

dRNA-seq libraries were prepared with Nanopore kits (SQK-RNA002, ONT; protocol version as released: September 13th, 2021) according to manufacturer’s instructions for both the control (poly(A)-selected) and unselected (poly(A) selection-omitted) libraries with the following modifications. For unselected libraries, input was increased from 500 nanograms of poly(A)-selected RNA to 5 micrograms total RNA. In all libraries, SuperScript IV (ThermoFisher Invitrogen, cat#18090010) was used rather than the ONT’s specification of SuperScript III (ThermoFisher Invitrogen, cat#18080051). No further modifications were introduced to the ONT dRNA-seq protocol.

To assess preparation yields, one microliter of each final library was quantified using the 1X HS DNA kit for Qubit (Thermofisher).

### Nanopore sequencing software, Basecalling, and alignment

All raw voltage traces were collected as FAST5 files using Oxford Nanopore Technologies’ software MinKNOW (versions 4.2.11 and 3.4.9). In order to minimize variability in basecalling and downstream analyses based on software versions, all libraries were reprocessed from FAST5s with the same pipeline as follows. Raw FAST5 files from MinKNOW were basecalled with Guppy (v6.0.1) in GPU mode using parameters: *guppy_basecaller -c rna_r9.4.1_70bps_hac.cfg*. Basecalled reads were aligned to *C. elegans* genome (WBCel235) using MiniMap2 (v2.17-r941) [13] with recommended settings for dRNA-seq: *minimap2 -x splice -uf -k14*. Additionally, parameter *–junc-bed* was used with a bed genome annotation file to provide minimap2 with splice junction information.

For libraries from Roach et al., 2020 [10], available FAST5 files were collected from the European Nucleotide Archive (ENA accession: PRJEB31791). All downstream processing of FAST5 files was identical to other processed libraries.

### Post-processing

Reads mapped to the genome were filtered using samtools (v1.10) [14] to ensure a single unique ‘best’ mapping position for each. Reads were assigned to genes and transcripts using two methods: (1) featureCounts (v2.0.0) [15] with the *--isLongRead* flag and (2) reads were assigned to annotated transcripts based on the mapping location of their 5′-most nucleotide. The combination of these methods were used because featureCounts was able to effectively identify reads that spanned the majority of a transcript, but faltered with abortive reads or partially degraded RNAs that did not contain sufficient information to determine transcript identity. The second method (based on read-end position) was able to identify the correct gene in such situations.

Nanopolish [9] was used with default parameters to assess poly(A) tail lengths in all sequenced libraries. For plots utilizing mean tail lengths as a summary statistic (Figs. 4 and 5), we restricted analyses to genes with 80 or more reads to minimize the effects of miscalled tails. Information from basecalling, mapping, gene assignment, and tail-length calling were consolidated into extended BAM format files [14] as additional tags. Reads were required to have successful mapping, gene assignment, and tail-length calling in order to be used for downstream analysis.

After read assignment to genes, information was consolidated into per gene summary statistics including mean and standard deviation for each: read length, tail length, and mapping quality. Read counts per gene were normalized to overall library depth to produce RPMs (Reads Per gene per Million), which was used for calculation of fold change between techniques.

### Plotting and visualizations

The Fig. 1 flow chart was produced using BioRender. All other figure visualizations were produced with Python using publicly available libraries: plotly (v5.3.0), seaborn (v0.11.0), pandas (v1.3.4), and matplotlib (v3.3.3). Statistical analyses were performed using the SciPy Python library (v1.5.4) using tests noted in text. Upset overlap plot (Fig. 3C) was generated with the python package upsetplot (v0.6.3) based on [8]. Gene decile groups based on change in tail length between selected and unselected libraries were calculated as projected along the diagonal of Fig. 5A. Decile assigned gene table is available as Supplementary Table 1.

## Supplementary Information

**Additional file 1.**

## Data Availability

Sequence data are available at SRA as PRJNA834154 (https://www.ncbi.nlm.nih.gov/Traces/study/?acc=PRJNA834154). The scripts used are also available from GitHub under MViscardi-UCSC/polyA-omission-manuscript.

## Abbreviations

ONT: Oxford Nanopore Technologies
dRNA-Seq: direct RNA-sequencing
CDS: Coding Sequence
CDF: Cumulative distribution function
RPM: Reads Per Million
KS Test: Kolmogorov–Smirnov test
ENA: European Nucleotide Archive
NGM: Nematode Growth Medium
RINe: RNA integrity number equivalent
